# Preliminary study on toxicological mechanism of golden cuttlefish (*Sepia esculenta*) larvae exposed to cd

**DOI:** 10.1186/s12864-023-09630-9

**Published:** 2023-08-30

**Authors:** Xiumei Liu, Xiaokai Bao, Jianmin Yang, Xibo Zhu, Zan Li

**Affiliations:** 1 College of Life Sciences, Yantai University, Yantai, 264005 China; 2 School of Agriculture, Ludong University, Yantai, 264025 China; 3 Fishery Technology Service Center of Lanshan District, Rizhao, 276800 China

**Keywords:** Cd, Neurotoxicity, Oxidative stress, Protein-protein interaction network, *Sepia esculenta*, Transcriptome

## Abstract

**Background:**

Cadmium (Cd) flows into the ocean with industrial and agricultural pollution and significantly affects the growth and development of economic cephalopods such as *Sepia esculenta*, *Amphioctopus fangsiao*, and *Loligo japonica*. As of now, the reasons why Cd affects the growth and development of *S. esculenta* are not yet clear.

**Results:**

In this study, transcriptome and four oxidation and toxicity indicators are used to analyze the toxicological mechanism of Cd-exposed *S. esculenta* larvae. Indicator results indicate that Cd induces oxidative stress and metal toxicity. Functional enrichment analysis results suggest that larval ion transport, cell adhesion, and some digestion and absorption processes are inhibited, and the cell function is damaged. Comprehensive analysis of protein-protein interaction network and Kyoto Encyclopedia of Genes and Genomes (KEGG) enrichment analysis was used to explore *S. esculenta* larval toxicological mechanisms, and we find that among the 20 identified key genes, 14 genes are associated with neurotoxicity. Most of them are down-regulated and enriched to the neuroactive ligand-receptor interaction signaling pathway, suggesting that larval nervous system might be destroyed, and the growth, development, and movement process are significantly affected after Cd exposure.

**Conclusions:**

*S. esculenta* larvae suffered severe oxidative damage after Cd exposure, which may inhibit digestion and absorption functions, and disrupt the stability of the nervous system. Our results lay a function for understanding larval toxicological mechanisms exposed to heavy metals, promoting the development of invertebrate environmental toxicology, and providing theoretical support for *S. esculenta* artificial culture.

**Supplementary Information:**

The online version contains supplementary material available at 10.1186/s12864-023-09630-9.

## Background

In recent years, heavy metals have been discharged into the ocean with pollutants generated by industries, agriculture, and healthcare, and have accumulated in large amounts in marine organisms, inducing growth inhibition and behavioral barriers [1–5]. Cadmium (Cd) is the most common heavy metal with high concentrations in the ocean [6–8]. It could increase reactive oxygen species (ROS) concentration of organisms in vivo to induce cell structure damage and biological dysfunction, including oxidative damage, DNA mutations, ion transport disorders, and protein destruction [9–13]. Meanwhile, Cd exposure can induce a large amount of Na^+^ loss in marine organisms and inhibit biological processes such as the storage of energy substances such as lipids and proteins [14–16].

*Sepia esculenta* is an economic cephalopod distributed along the eastern coast of China [17–20]. Compared to other marine organisms, *S. esculenta* larvae are relatively fragile, and adverse environments can significantly inhibit their development [21–24]. In previous studies, Cd has been found to reduce the hatchability of larvae, induce oxidative damage and toxic reaction, and promote the death of larvae [25–27]. The toxicological mechanism of Cd-exposed golden cuttlefish *S. esculenta* larvae is still unclear.

RNA-Seq is an important method for exploring biological differences at the molecular level [28–30]. In addition to model species, it has been applied to non-model mollusks in recent years [31, 32]. Recently, RNA-Seq was used to explore the toxicological mechanism of marine organisms exposed to heavy metals. Shi et al. found that the ATP-binding cassette transporters superfamily of *Crassostrea angulate* plays an important role in maintaining Cu concentration, regulating apolipoprotein transport, and clearing excess Cu [33]. Li and Wang showed that high concentration of Cd exposure will induce the disorder of DNA replication and transcription process, thus affecting the growth of oysters [34]. Zhao et al. indicated that Cd exposure could affect proteasome and oxidative physiology-related signaling pathways, thereby inducing oxidative stress in the kidney of *Chlamys farreri* [35]. Hence, Cd-exposed *S. esculenta* larval toxicological mechanisms can be explored through RNA-Seq.

In our research, we used superoxide dismutase (SOD), glutathione S-transferase (GST), metallothionein (MTs), and malondialdehyde (MDA) to assess the molecular responses of exposed *S. esculenta*. Meanwhile, we used functional enrichments to identify key terms and pathways. In addition, a comprehensive analysis of Kyoto Encyclopedia of Genes and Genomes (KEGG) and Protein-protein interaction (PPI) network was used to study Cd-exposed *S. esculenta* larval toxicological mechanisms. Our results laid a function for exploring the effects of heavy metal pollution on the biological processes of cephalopods. At the same time, the results further deepen the understanding of changes in oxidative and toxicity of mollusks after environmental stress, promoting the development of the mollusk breeding industry.

## Materials and methods

### Larval collection and exposure

In this study, larvae were hatched 28 days after the eggs were laid. After pre-experiment, it was confirmed that the LC50 (semilethal concentration) for 24 h under Cd exposure conditions was 64.7 ug/L. Then, we divided 200 larvae (mantle length (body size) = 6.2 ± 0.2 mm; weight = 62.8 ± 8.2 mg) into two groups, including the control group (C) and the 50 ug/L Cd-exposed group (Cd). Finally, larvae in both groups grew for 24 h in plastic barrels, respectively, and were collected at 0 h (C_0h), 4 h (C_4h and Cd_4h), and 24 h (C_24h and Cd_24h). We used liquid nitrogen to freeze the larvae quickly and collected them for preservation in a cryopreservation tube.

### Assay of antioxidant enzymes and toxicity

SOD, MDA, and GST activities of *S. esculenta* larvae were measured using kits produced by Nanjing Jiancheng Bioengineering Institute, and MTs activity was measured using the kits produced by Shanghai Enzyme-linked Biotechnology. Their measuring methods were described by Marklund and Marklund [36], Eren [37], Satoh [38], and Penicaud, et al. [39], respectively.

### RNA extraction and sequencing

We used the TRI Reagent method [40] with the manufacturer’s protocol to extract total RNA and identified the integrity using Agilent 2100 bioanalyzer [41], and the whole larva was used for RNA extraction. We selected three larvae from each group and mixed their RNA based on molar masses into a biological repeat, and repeated this process three times. We used these three replicates to construct the library [42]. Raw reads were sequenced by Illumina NovaSeq 6000 (Illumina, USA), and low-quality reads were removed. Then, HISAT2 was used to map obtained clean reads to the reference genome of our laboratory (unpublished).

### DEG identification and functional enrichment analyses

In each time point, DESeq2 thresholds *p*-value ≤ 0.05 and fold change ≥ 1.5 was used to identify differentially expressed genes (DEGs) [43]. Then, DEGs were enriched into terms and pathways using DAVID to identify their functions [44].

### Identification and validation of key and genes

Genes enriched in significant signaling pathways were used to construct a PPI network using STRING [45]. The key and hub genes were identified based on KEGG enrichment and PPI network analysis, and the accuracy of key and hub genes was verified using qRT-PCR [46]. Table S1 shows their primer sequences.

## Results

### Oxidative stress and metal toxicity

**Fig. 1:**
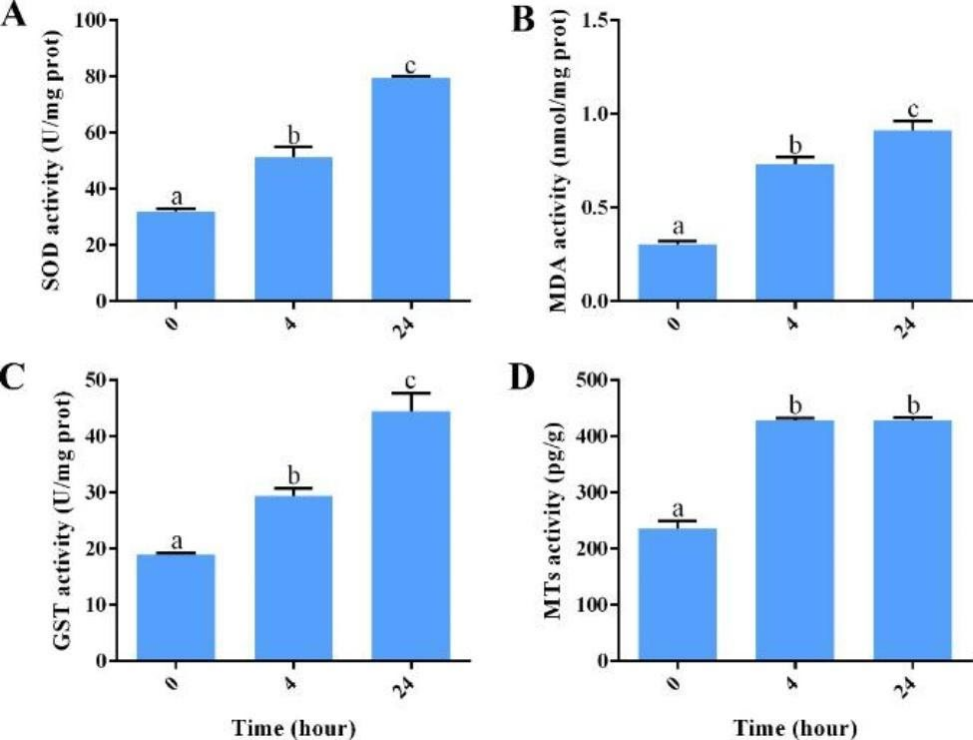
Results of SOD (**A**), MDA (**B**), GST (**C**), and MTs (**D**) activities. a, b, and c stand for significant differences between exposure times

Figure 1 shows that the activities of SOD, MDA, GST, and MTs increase significantly after 4 h exposure. Among these, the activity of MTs increases significantly at 4 h of exposure and does not change significantly within 4 to 24 h. SOD, MDA, and GST activities exhibit a significant dose-dependent increase (*p*-value < 0.05) with the extension of exposure time.

### Sequencing results and DEGs expression

**Fig. 2:**
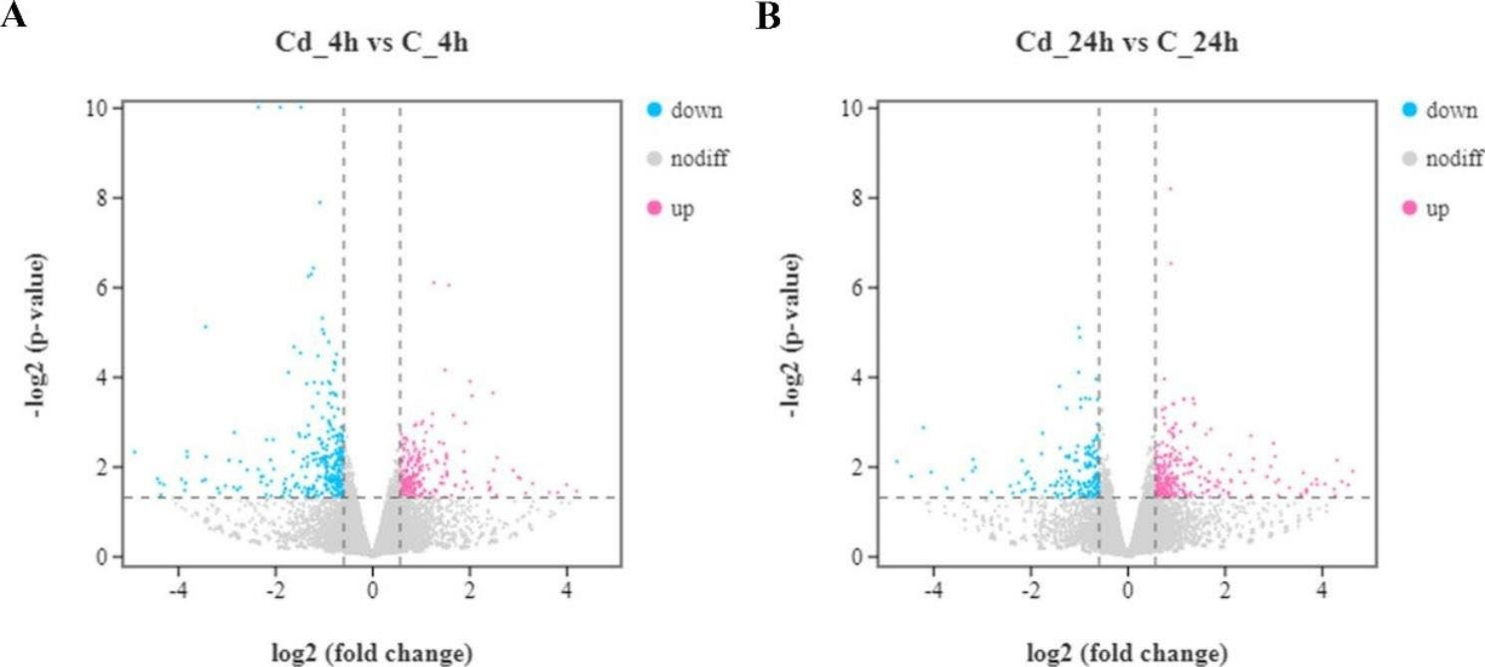
Expression difference of DEGs. (**A**) DEGs expression distribution at 4 h exposure. Up-regulated, down-regulated, and non-regulated genes are indicated by pink, blue, and grey dots, respectively. (**B**) DEGs expression distribution at 24 h exposure

**Fig. 3:**
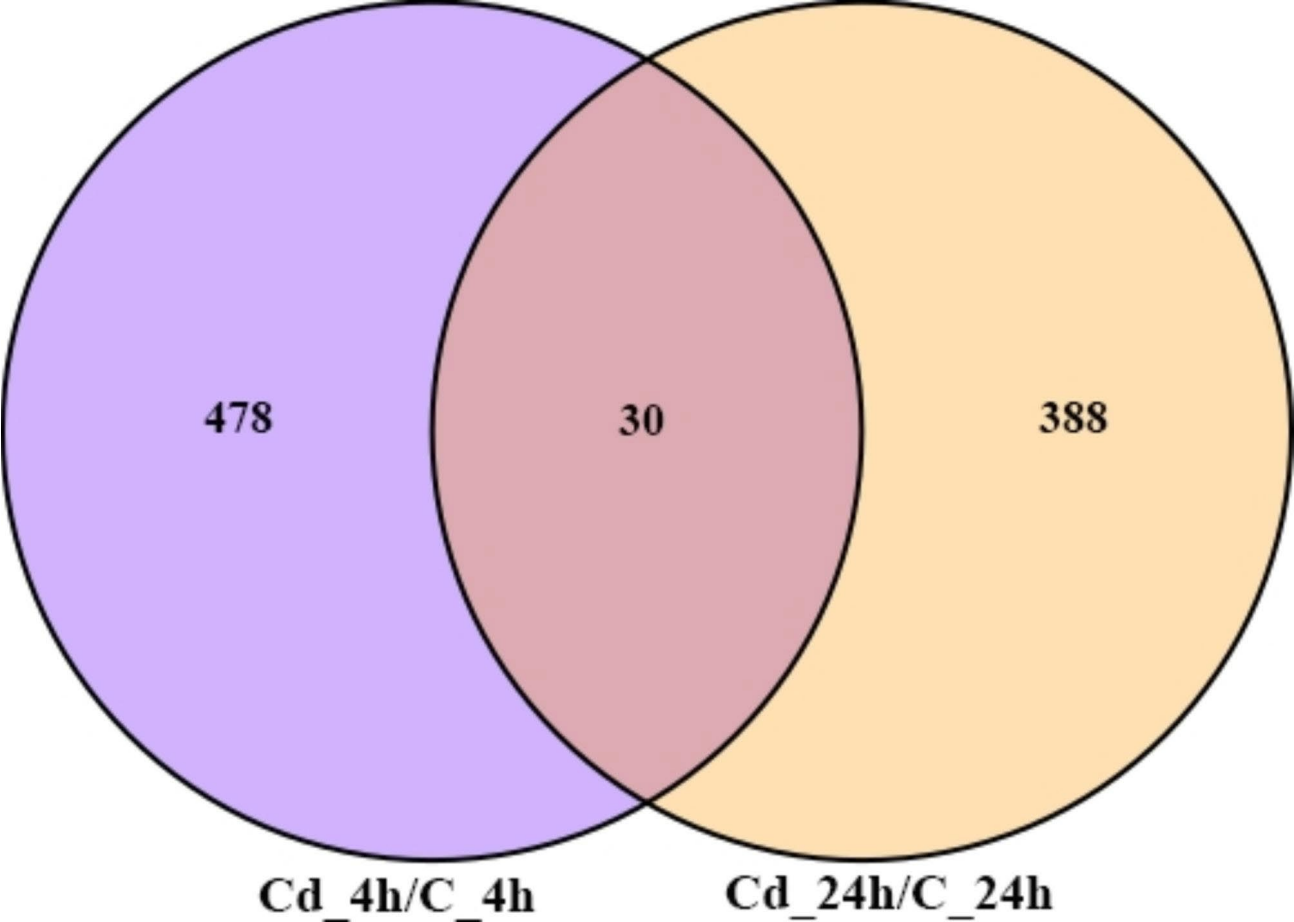
DEG distributions between two time points. Different colors represent different DEGs expression distributions

**Fig. 4:**
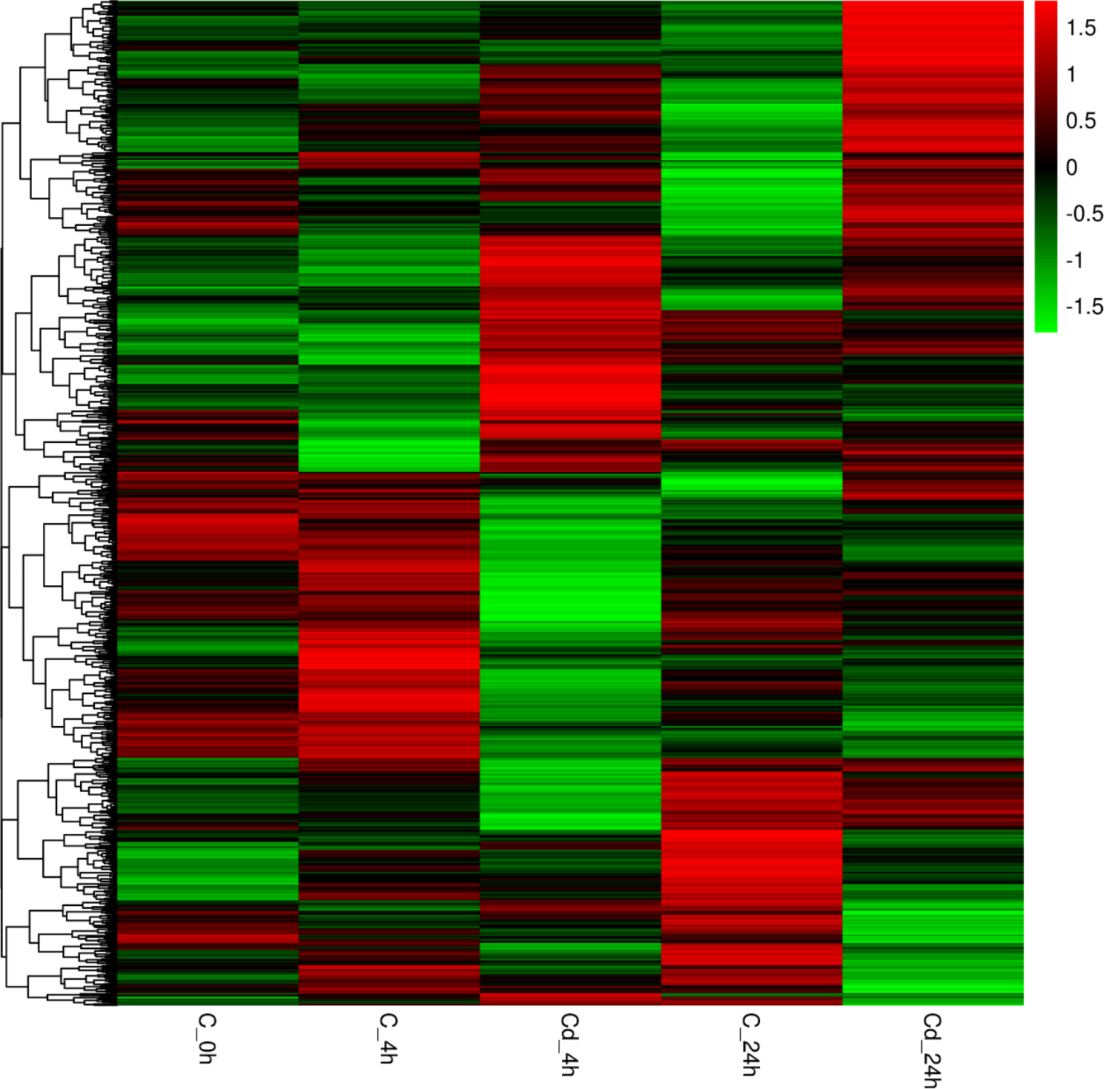
Expression clustering of DEGs. A row indicates expressions of a DEG in each group; each column represents the expression amount of all DEGs in a group

An average of 43,963,462 raw reads and 43,385,588 (98.69%) clean reads are sequenced (Table S2). The Volcano plot results reveal that at 4 h exposure, 207 genes are up-regulated, and 301 genes are down-regulated. At 24 h exposure, 257 genes are up-regulated, and 161 genes are down-regulated (Fig. 2). In addition, Fig. 3 illustrates that there are 896 DEGs within 24 h of exposure, and 30 of these DEGs are expressed differently at two time points. The expression distribution of the DEGs is presented in the heatmap (Fig. 4).

### Functional enrichment of DEGs

**Fig. 5:**
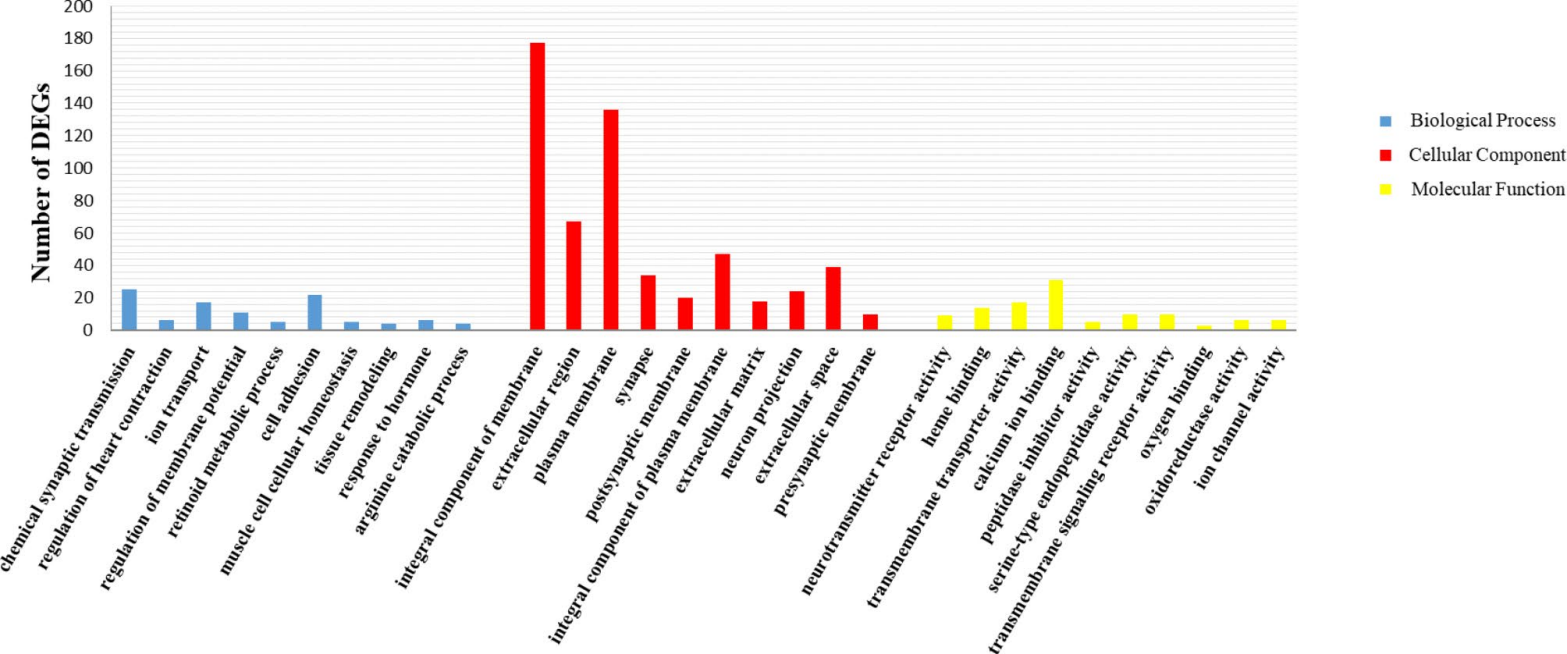
Top 10 significant GO terms in each classification

**Fig. 6:**
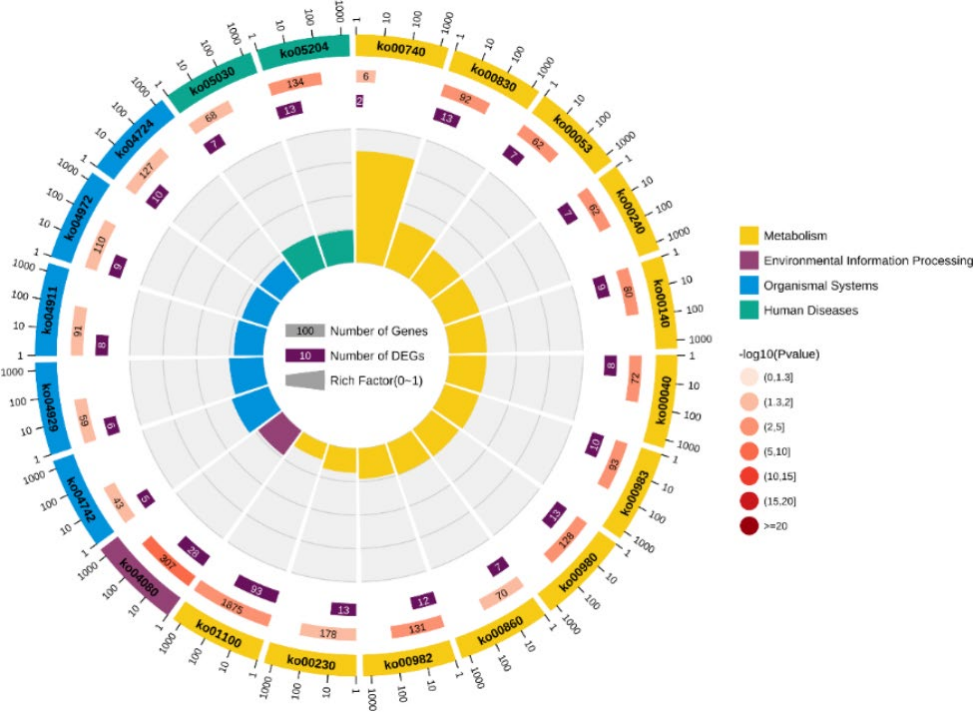
Top 20 level-2 KEGG signaling pathways results. The outermost circle represents the enriched level-2 KEGG pathways, and different colors stand for different classes; the second outer circle indicates the number of genes enriched into the pathway in the background gene set; the third circle represents the gene numbers enriched into the pathway in the input gene set; rich factor stands for the ratio of the number of genes in the input gene set enriched in the pathway to enriched gene numbers in the background gene set

This study enriched 76 significant GO terms (Fig. 5). Among them, chemical synaptic transmission, cell adhesion, and other terms are important for regulating oxidative stress. KEGG enrichment analysis suggests that level-2 KEGG signaling pathways regulating oxidation and toxic reactions such as signal transduction and cell growth and death are enriched (Fig. 6). And the enrichment of signaling pathways such as cAMP signaling pathway, PI3K-Akt signaling pathway, and lysosome signaling pathway indicates that larval oxidative stress and toxicity might be significantly induced after Cd exposure.

**Table 1:** Significant level-3 KEGG signaling pathways statistics

Pathways	Number of DEGs	GeneRatio (%)
Arginine biosynthesis	2	0.90
Autophagy - animal	2	0.90
cAMP signaling pathway	6	2.71
Carbohydrate digestion and absorption	3	1.36
Carbon metabolism	2	0.90
Chemical carcinogenesis - DNA adducts	6	2.71
Chemical carcinogenesis - receptor activation	4	1.81
Cholesterol metabolism	2	0.90
Estrogen signaling pathway	3	1.36
GnRH secretion	5	2.26
Lysosome	6	2.71
Natural killer cell mediated cytotoxicity	2	0.90
Neuroactive ligand-receptor interaction	19	8.60
Phospholipase D signaling pathway	3	1.36
PI3K-Akt signaling pathway	4	1.81
Protein digestion and absorption	3	1.36
Relaxin signaling pathway	3	1.36
Retinol metabolism	6	2.71

### Key and hub genes analysis

**Fig. 7:**
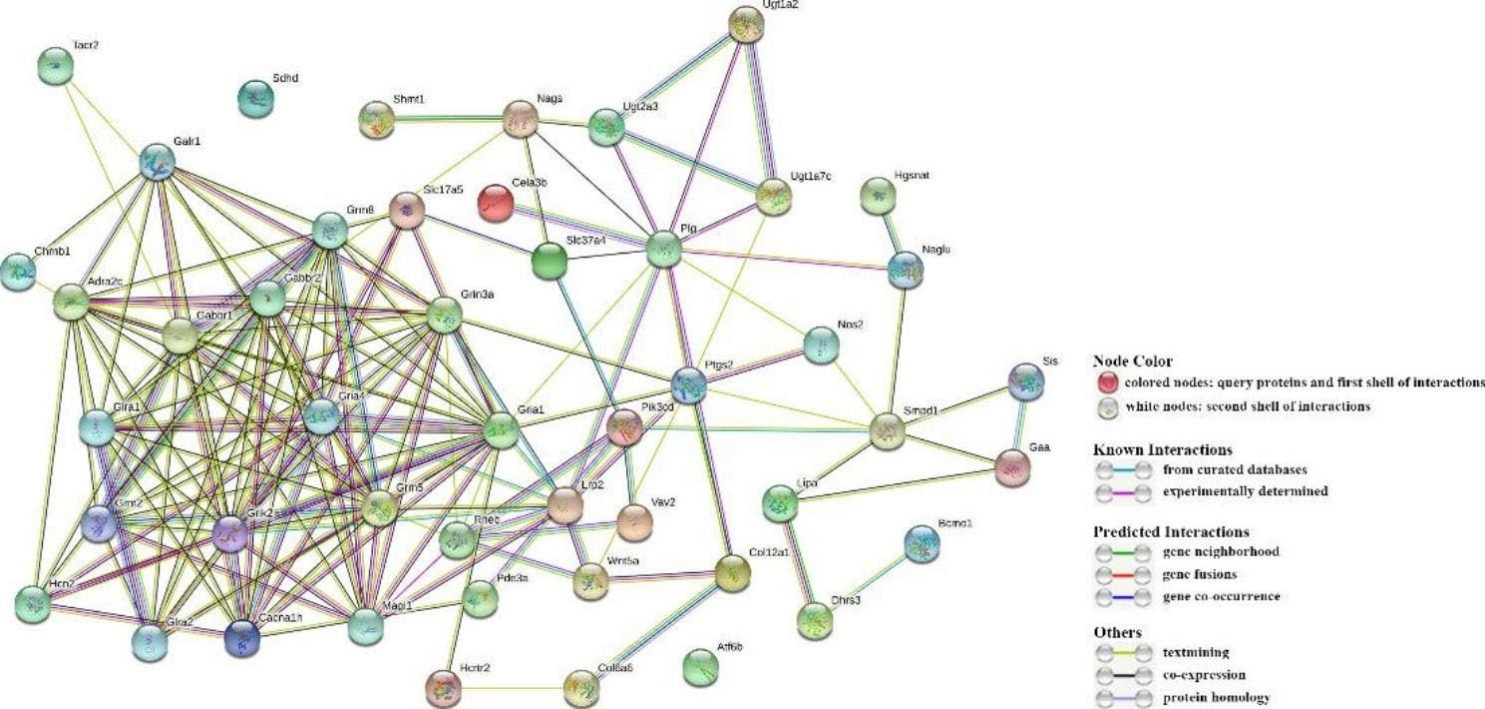
The PPI network. The dots stand for proteins, and the connection represents the interaction between genes

**Fig. 8:**
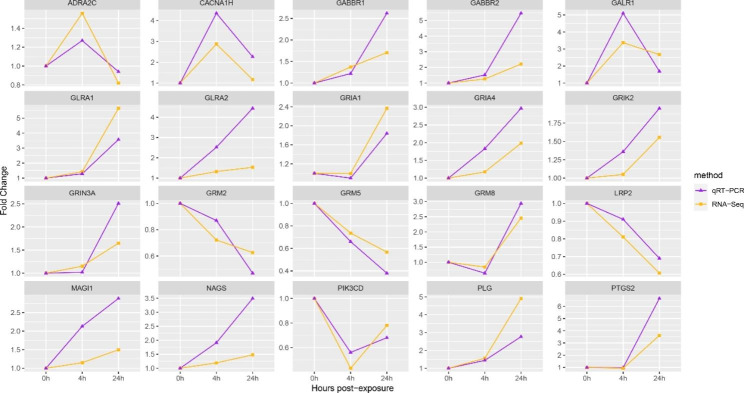
Gene expression verification (qRT-PCR). The abscissa represents Cd exposure time; the ordinate stands for fold change

We used 48 DEGs enriched in pathways listed in Table 1 to construct the PPI network (Fig. 7). Table 2 shows relevant parameters. Among the network, three hub genes interacting with the most genes or involving in the most pathways in Table 1, including GRIA1, GRIA4, and GRIK2, are identified; and 17 key genes with higher protein interaction numbers or higher KEGG pathway participation numbers are identified at the same time (Table 3). qRT-PCR result suggests that DEGs in Table 3 are single products, and RNA-Seq results are accurate (Fig. 8).

**Table 2:** The network parameters

Network statistics	
Number of nodes	48
Number of edges	168
Average node degree	7
Clustering coefficient	0.578
Expected number of edges	78
PPI enrichment *p*-value	1.0E-16

**Table 3:** Summary of key and hub DEGs.

Gene name (abbreviation)	Gene name (official full name)	Number of KEGG signaling pathways	Number of protein-protein interactions
*ADRA2C*	adrenoceptor alpha 2 C	1	14
*CACNA1H*	calcium voltage-gated channel subunit alpha1 H	1	12
*GABBR1*	gamma-aminobutyric acid type B receptor subunit 1	2	16
*GABBR2*	gamma-aminobutyric acid type B receptor subunit 2	4	16
*GALR1*	galanin receptor 1	1	10
*GLRA1*	glycine receptor alpha 1	1	12
*GLRA2*	glycine receptor alpha 2	1	9
*GRIA1*	glutamate ionotropic receptor AMPA type subunit 1	1	21
*GRIA4*	glutamate ionotropic receptor AMPA type subunit 4	2	17
*GRIK2*	glutamate ionotropic receptor kainate type subunit 2	2	17
*GRIN3A*	glutamate ionotropic receptor NMDA type subunit 3 A	2	16
*GRM2*	glutamate metabotropic receptor 2	2	11
*GRM5*	glutamate metabotropic receptor 5	2	16
*GRM8*	glutamate metabotropic receptor 8	2	15
*LRP2*	LDL receptor related protein 2	1	10
*MAGI1*	membrane associated guanylate kinase, WW and PDZ domain containing 1	1	11
*NAGS*	N-acetylglutamate synthase	1	5
*PIK3CD*	phosphatidylinositol-4,5-bisphosphate 3-kinase catalytic subunit delta	8	5
*PLG*	plasminogen	1	12
*PTGS2*	prostaglandin-endoperoxide synthase 2	1	9

## Discussion

### Purpose and significance

This study aims to reveal the effect of Cd acute exposure on the molecular mechanisms of *S. esculenta* larvae. In previous studies, oxidative stress, immune response, and metabolic mechanisms of cephalopods exposed to Cd were studied based on multiple experiments [39, 47, 48]. For example, Cd inhibits the energy metabolism of *Sepia pharaonis* by inhibiting the expression of arginine kinase [47]. Cd exposure induces oxidative stress and regulates the immune response of *Sepiella maindroni* by regulating the expression of HSP70 [48]. We supplemented the transcriptome analysis on the basis of experimentally exploring the oxidation and toxicity mechanisms of *S. esculenta* larvae to further reveal the larval response to Cd at the molecular level.

### Biochemical and molecular biomarkers analysis

Cd has significant effects on ROS concentration in organisms [49, 50]. When ROS concentration is stable at normal level, it involves and regulates cell processes such as proliferation and differentiation, immune signal transduction, and transcription factor expression and plays an important role in maintaining biological life activities [51, 52]. However, excessive concentration of ROS will induce DNA strand breakage, cell function and tissue structure damage, lipid peroxidation, and other oxidative damage [53, 54]. To mitigate the adverse effects caused by oxidative damage, the organism will activate the antioxidant defense system to deal with oxidative stress, which promotes the synthesis and expression of SOD and some other antioxidant substances to remove excess ROS [55, 56]. SOD is the most important antioxidant enzyme defense system to eliminate ROS. It can catalyze superoxide anion free radicals into H_2_O_2_, which is then decomposed into water by other antioxidant enzymes to resist the oxidative stress process [55, 57]. MDA is the main product of polyunsaturated fatty acid peroxidation, which is used to identify biological lipid peroxidation degree [58–60]. In our study, with the increase of exposure time, the activities of SOD and MDA continue to increase, suggesting that tissues and organs of *S. esculenta* larvae exposed to Cd are seriously damaged by oxidation, and the integrity of membrane-related biomolecules is damaged. Meanwhile, Cd exposure can induce metal toxicity. GST is a key enzyme that regulates cell detoxification, and reduces metal toxicity by regulating GSH binding to heavy metals [61, 62]. MTs can catalyze heavy metals into metal-MT complexes to reduce the content of heavy metals in cells and reduce the damage of heavy metals to proteins and tissues by seizing heavy metal ions combined with other proteins [63, 64]. Based on our results, we speculate that Cd induces significant metal toxicity, and GST and MTs reduce toxicity by combining the excess heavy metals in *S. esculenta* larvae. The significant changes of SOD, MDA, GST, and MTs activities show that Cd induces oxidative stress and increases the activity of antioxidant enzymes, leading to oxidative damage in *S. esculenta* larvae from the effects of Cd exposure.

### GO functional enrichment analysis

Ion transport, cell adhesion, and other processes are significantly inhibited in Cd-exposed larvae. Multiple terms regulating ion binding and transportation such as ion transport, calcium ion binding, and ion channel activity are significantly enriched. Cd exposure affected almost all ion transport and inhibited ion binding, thereby destroying ion homeostasis [65–67]. And the ion disorder could significantly affect the metabolic processes of organisms [68, 69]. We preliminarily speculate that Cd might affect the ion homeostasis of larvae by inhibiting ion transport and binding, and inducing metabolic toxicity to inhibit larval growth and development. Down-regulation of DEGs enriched in cell adhesion and muscle cell cellular homeostasis terms indicates that larval cell functions might be inhibited after Cd exposure. Among them, the destruction of cell adhesion function could inhibit the immune functions of larvae and reduce their immunity [70]. And the disorder of muscle cells might induce motor dysfunction in *S. esculenta* larvae and inhibit their swimming ability [71]. The enrichment of oxygen binding and oxidoreductase activity terms might be the result of Cd promoting oxidative stress and inducing oxidative damage in *S. esculenta* larvae, which is consistent with the results of up-regulated SOD and MDA activities in larvae exposed to Cd in this study [72, 73].

### KEGG functional enrichment analysis

A large number of KEGG signaling pathways were enriched after Cd exposure. The results show that *S. esculenta* larvae may be adversely affected in multiple aspects such as digestion and absorption and cell functions.

#### Dysfunction of digestion and absorption

Digestion and absorption are essential processes to maintain the life activities of organisms [74]. Disorder of this process will significantly reduce the efficiency of energy acquisition and inhibit the absorption and transport of nutrients [75, 76]. In this study, protein digestion and absorption signaling pathway and carbohydrate digestion and absorption signaling pathway were enriched, and most DEGs enriched in pathways were down-regulated. We speculate that digestion and absorption process of carbohydrates and proteins are inhibited after Cd exposure. The down-regulation of genes enriched in relevant pathways maintaining cell and extracellular matrix structure and regulating cell functions, including collagen type VI alpha 6 chain (COL6A6) and collagen type XII alpha 1 chain (COL12A1), might be the result of Cd-induced oxidative damage to cells in the digestive system [77–79]. This result is consistent with the increase in SOD and MDA activities in this study. In addition, the up-regulation of solute carrier family 37 member 4 (SLC37A4) indicates that *S. esculenta* larvae might resist oxidative damage induced by Cd by promoting glucose metabolism and energy production to promote antioxidant reaction [80, 81]. In conclusion, we preliminarily speculate that Cd exposure might inhibit larval absorption and digestion process by inducing oxidative damage to cells. The reason for the up-regulation of SLC37A4 still needs to be elucidated in follow-up studies.

#### Cell functions and oxidative damage

The PI3K-AKT is a core signaling pathway regulating cellular functions such as growth, proliferation, migration, and survival [82, 83]. It plays a significant role in antioxidant reactions [84]. Previous studies have shown that oxidative stress induces tissue peroxidation, inhibits cell growth and proliferation, and induces oxidative damage to cells [84, 85]. While regulating cell function, activation of PI3K-AKT signaling pathway promotes the expression of antioxidant genes and regulates apoptosis to reduce oxidative damage to cells [86, 87]. At the same time, PI3K-AKT signaling pathway acts a key part in the repair of DNA damage caused by oxidative stress and regulation of protein synthesis [88]. In this study, the activities of two antioxidant enzymes are significantly up-regulated, suggesting that Cd may induce severe oxidative stress. And PI3K-AKT signaling pathway might reduce oxidative damage of *S. esculenta* larval cells by regulating cell apoptosis, DNA repair, and protein synthesis.

### Cd-induced neurotoxicity based on analysis of key and hub genes

The nervous system is the most important system that regulates all physiological functions of organisms [89, 90]. It can sense and respond to changes in the internal and external environment and play a leading role in maintaining biological breathing, feeding, movement, and other life processes [91, 92]. Among the 20 key and hub genes with a high number of protein interactions or KEGG pathway participation, 14 genes, including hub gene, have been identified to regulate the stability of the nervous system in previous studies, and these genes are enriched in neuroactive ligand-receptor interaction signaling pathway that regulates nervous system functions [93]. This result suggests that Cd might significantly affect larval nervous system and induce neurotoxicity. GABBR is a receptor for the key inhibitory neurotransmitter GABA in the central nervous system and regulates the inhibition of neurons [94, 95]. Meanwhile, as an important regulator of the GABAergic system, GABBR acts a crucial part in regulating the functions of the brain [96, 97]. GABBR can be divided into two subunits, GABBR1 and GABBR2, which play key roles in neurotransmitter transmission [94, 98]. The most important function of GABBR1 and GABBR2 is to regulate synaptic transmission, and their overexpression inhibits presynaptic Ca channel functions such as Ca^2+^ influx, thereby inhibiting synaptic transmission in the central nervous system and inducing neurotoxicity [95, 99]. It is worth noting that Tsentsevitsky and Petrov have found in previous studies that Cd exposure reversibly blocks the voltage-gated Ca^2+^ channels, promoting Cd absorption [100]. The significant up-regulation of GABBRs in the present study indicates that synaptic transmission function in Cd-exposed *S. esculenta* larvae might be inhibited, and the absorption rate of Cd may be increased, promoting the toxicity of Cd and reducing the stability of the nervous system. Glutamate receptors (GlyRs) are another protein that regulates inhibitory neurotransmission in the central nervous system [101, 102]. GLRA1 and GLRA2, subunit encoding inhibitory glycine receptors, were found in recent years to be mainly used to regulate the stability of glycinergic inhibitory synapse functions [103, 104]. In fish, these two genes regulate the transmission of inhibitory neurotransmitters and play central roles in the regulation of motor functions. And the abnormal expression of GLRA1 and GLRA2 induces motor coordination disorder and inhibits swimming, breathing, eating and other exercise processes [103–105]. In this research, GLRA1 and GLRA2 are up-regulated, indicating that Cd might damage larval motor nerves and inhibit various motor processes. Hub genes, including GRIA1, GRIA4, and GRIK2, are identified to further explore changes in larval neural functions after Cd exposure. AMPA receptor is a glutamate gated cation channel which has been widely concerned because of its important neural regulation function [106, 107]. As the genes encoding the AMPA receptor components GluA1 and GluA4, GRIA1 and GRIA4 play significant roles in regulating neural functions such as synaptic development and transmission [108, 109]. And their abnormal expressions could cause the disorder of synaptic functions that induce neurodevelopmental and motor dysfunction as well as impaired memory ability and behavioral phenotypes [109, 110]. As a gene encoding another glutamate ionotropic receptor KARs in the central nervous system, GRIK2 has similar functions to GRIA1 and GRIA4 such as the regulation of neurotransmitter release and synaptic plasticity and induces neurodevelopmental disorders and decreased exercise vitality when abnormally expressed [111, 112]. Three hub genes are significantly up-regulated in this study. Based on previous studies on these three hub genes, we preliminarily speculate that Cd might significantly inhibit the development of the nervous system of *S. esculenta* larvae and induce neurological dysfunction. In addition, the functions of the remaining neuro-related key genes, including ADRA2C, GALR1, GRIN3A, GRM2, GRM5, GRM8, and PLG, such as neuron and synaptic transmission, maintenance of nervous system homeostasis, and regulation of ion transport have also been found in previous studies [113–119]. In conclusion, we preliminarily speculate that the larval nervous system has significant changes after Cd exposure. Cd might inhibit the development of nervous system and induce neurotoxicity in larvae by inhibiting neuronal and synaptic transmission, hindering ion transport, and reducing motor coordination ability and significantly inhibiting larval growth and development. However, these genes have not been studied in marine mollusks, and their functions in *S. esculenta* are still unclear. High concentrations of Cd in coastal areas induce oxidative damage and metal toxicity, inhibit the growth and development of marine organisms, reduce larval hatching and survival rates, and seriously hinder the artificial breeding of marine organisms. Therefore, the neurotoxicity induced by Cd in *S. esculenta* larvae needs to be further explored in the future.

## Conclusion

We use a combination of experimental index measurement and innovative transcriptome analysis to explore Cd-exposed *S. esculenta* larval molecular mechanisms. The results indicate that biochemical and molecular biomarkers, including SOD, MDA, GST, and MTs, show significant changes after Cd exposure, indicating that Cd might induce peroxidation in larval tissues of *S. esculenta* and trigger toxic reactions. The results of transcriptome analysis showed that the digestion and absorption functions of larvae were inhibited and cells might be severely damaged by oxidation. In addition, the most important point is that larval nervous system might be destroyed, and the neural development and motor coordination of larvae might be significantly inhibited. This study preliminarily reveals Cd-exposed larval toxicological mechanisms, laying a foundation for understanding the response mechanism of cephalopods to heavy metal exposure deeply, providing theoretical support for the artificial cultivation of *S. esculenta*, and promoting the development of cephalopod aquaculture.

### Electronic supplementary material

Below is the link to the electronic supplementary material.

Supplementary Material 1

Supplementary Material 2

## Data Availability

The datasets generated and analysed during the current study are available in the NCBI repository, accession number SRR19578101, SRR19578102, SRR19578103, SRR19578104, SRR19578105, SRR19578106, SRR19578107, SRR19578113, SRR19578114, SRR20545810, SRR20545811, SRR20545812, SRR20545813, SRR20545816, SRR20545817 at the following link: ncbi.nlm.nih.gov/Traces/study/?acc = PRJNA844162&o = library_name_s%3Aa.
