# Supplementary material for: Preliminary study on toxicological mechanism of golden cuttlefish (Sepia esculenta) larvae exposed to cd
Source: BMC Genomics. 2023 Aug 30;24:503. doi: 10.1186/s12864-023-09630-9 (PMC10466719; doi:10.1186/s12864-023-09630-9)
Supplement: Supplementary file 1 — Supplementary Material 1 [file 12864_2023_9630_MOESM1_ESM.docx]

**Table S1.** List of primers used for quantitative RT-PCR validation.

| Gene name | Forward primer (5’-3’) | TM(°C) | Reverse primer (5’-3’) | TM(°C) | Amplicon length (bp) |
| --- | --- | --- | --- | --- | --- |
| *ADRA2C* | GGACGCTCAACACTACATTC | 60 | GTCTTGTGGTCCTGCTTTC | 60 | 111 |
| *CACNA1H* | GGAATGTATCGGCCATGTT | 60 | GTCCCATGGCAATCATCTT | 60 | 127 |
| *GABBR1* | GAAGGTTATGCCTGGTTTCT | 60 | TCTTGAGGCCGGTTATCT | 60 | 101 |
| *GABBR2* | TTGCCTGGGAAATACGTAAC | 60 | CACAGGCCACTCCTTTATTAG | 60 | 134 |
| *GALR1* | AGACGTGGACTCCTACAA | 59 | GACGACGGTGGGTATAAAG | 59 | 106 |
| *GLRA1* | CCTTGTCAACGTCCAACTT | 60 | GAGCCTTTCATCTCTCCATTC | 60 | 109 |
| *GLRA2* | GACAGACGAAGAGCAGAAAG | 60 | CGCTTGCTTGTTGTTTGAG | 60 | 102 |
| *GRIA1* | GTTCCGCAATTCGAGATACT | 60 | ATCCACATTGGCCTTTCTC | 60 | 108 |
| *GRIA4* | TATCACCGAGGTCCATATCC | 60 | GTGAGGAAAGCTGCTAAGTT | 60 | 103 |
| *GRIK2* | CCGTCCGTGTATGTTTCTT | 60 | TGACGTTGAACCTGGTAATC | 60 | 107 |
| *GRIN3A* | CCCTCAGTGTCACCATTAAC | 60 | GCCATCATCGAACAATCCT | 60 | 121 |
| *GRM2* | GGCTATTGCCGATTACTCTAC | 60 | GTACTGTCCGCATGGTATAAG | 60 | 110 |
| *GRM5* | ACCCGTGGTTAATGGAATG | 60 | CGTTGCTACTGTCGAGTTAC | 60 | 101 |
| *GRM8* | AGCGTATGATCCGAGAGAA | 60 | CACGGTTTGCCCAGTAAT | 60 | 128 |
| *LRP2* | ATGCAACACTGGCCTTAC | 60 | GGCTTCAGGGTGATCATTAG | 60 | 124 |
| *MAGI1* | ACCAGGAGGCCATTGATA | 60 | AGTTCGATTTGTGGGAGAAG | 60 | 116 |
| *NAGS* | AGATGTTGTGGGCTTGTG | 60 | GCTCCAACTTCCTTCAGATT | 60 | 110 |
| *PIK3CD* | GGTGTTGTACGTGATAGAGTG | 60 | CGGCTGCTGGAACAAATA | 60 | 131 |
| *PLG* | GTCACTGCCCGATTATTACC | 60 | CCGAGCTGCTACATATTCTTC | 60 | 126 |
| *PTGS2* | CCACAGCCCAGACAATTTA | 60 | CTGCTTGCTCGTGTAGATAG | 60 | 139 |
