# Supplementary material for: Preliminary study on toxicological mechanism of golden cuttlefish (Sepia esculenta) larvae exposed to cd
Source: BMC Genomics. 2023 Aug 30;24:503. doi: 10.1186/s12864-023-09630-9 (PMC10466719; doi:10.1186/s12864-023-09630-9)
Supplement: Supplementary file 2 — Supplementary Material 2 [file 12864_2023_9630_MOESM2_ESM.docx]

**Table S2.** Sequencing quality and mapping results.

| Samples | Raw reads | Clean reads | Q20 (%) | Q30 (%) | GC (%) | Mapping rate (%) |
| --- | --- | --- | --- | --- | --- | --- |
| C_0h_1 | 44,822,088 | 44,401,358 | 97.42 | 93.02 | 39.89 | 87.71 |
| C_0h_2 | 46,604,268 | 46,067,346 | 97.39 | 92.97 | 38.64 | 87.25 |
| C_0h_3 | 42,199,716 | 41,745,596 | 97.08 | 92.31 | 39.10 | 86.13 |
| C_4h_1 | 42,594,570 | 42,050,900 | 97.56 | 93.35 | 39.79 | 88.28 |
| C_4h_2 | 45,122,216 | 44,583,624 | 97.37 | 92.89 | 40.01 | 87.80 |
| C_4h_3 | 43,910,186 | 43,339,204 | 97.44 | 93.00 | 39.72 | 87.67 |
| Cd_4h_1 | 44,237,100 | 43,609,904 | 97.45 | 93.06 | 40.65 | 88.39 |
| Cd_4h_2 | 45,963,126 | 45,180,404 | 97.49 | 93.07 | 39.24 | 87.38 |
| Cd_4h_3 | 45,268,732 | 44,022,418 | 97.58 | 93.27 | 40.04 | 87.88 |
| C_24h_1 | 45,374,672 | 44,918,056 | 97.51 | 93.17 | 39.82 | 88.32 |
| C_24h_2 | 40,894,638 | 40,402,580 | 97.59 | 93.30 | 38.92 | 88.10 |
| C_24h_3 | 42,664,646 | 42,060,564 | 97.65 | 93.45 | 38.74 | 87.88 |
| Cd_24h_1 | 42,041,608 | 41,591,474 | 97.52 | 93.15 | 39.70 | 87.88 |
| Cd_24h_2 | 43,777,446 | 43,297,372 | 96.91 | 91.51 | 38.92 | 87.45 |
| Cd_24h_3 | 43,976,924 | 43,513,024 | 97.32 | 92.66 | 38.89 | 87.33 |
